# The NSCLC immunotherapy response predicted by tumor-infiltrating T cells via a non-invasive radiomic approach

**DOI:** 10.3389/fimmu.2024.1379812

**Published:** 2024-09-09

**Authors:** Jie Min, Fei Dong, Yongyuan Chen, Wenshan Li, Yimin Wu, Yanbin Tan, Fan Yang, Pin Wu, Ying Chai

**Affiliations:** ^1^ Department of Radiology, The Second Affiliated Hospital, Zhejiang University School of Medicine, Hangzhou, Zhejiang, China; ^2^ Department of Thoracic Surgery, The Second Affiliated Hospital, Zhejiang University School of Medicine, Hangzhou, Zhejiang, China

**Keywords:** non-small cell lung cancer, tumor immune microenvironment, immune checkpoint inhibitors, immunotherapy, radiomic

## Abstract

**Introductions:**

Identifying patients with non-small cell lung cancer (NSCLC) who are optimal candidates for immunotherapy is a cornerstone in clinical decision-making. The tumor immune microenvironment (TIME) is intricately linked with both the prognosis of the malignancy and the efficacy of immunotherapeutic interventions. CD8+ T cells, and more specifically, tissue-resident memory CD8+ T cells [CD8+ tissue-resident memory T (TRM) cells] are postulated to be pivotal in orchestrating the immune system's assault on tumor cells. Nevertheless, the accurate quantification of immune cell infiltration—and by extension, the prediction of immunotherapeutic efficacy—remains a significant scientific frontier.

**Methods:**

In this study, we introduce a cutting-edge non-invasive radiomic model, grounded in TIME markers (CD3+ T, CD8+ T, and CD8+ TRM cells), to infer the levels of immune cell infiltration in NSCLC patients receiving immune checkpoint inhibitors and ultimately predict their response to immunotherapy. Data from patients who had surgical resections (cohort 1) were employed to construct a radiomic model capable of predicting the TIME. This model was then applied to forecast the TIME for patients under immunotherapy (cohort 2). Conclusively, the study delved into the association between the predicted TIME from the radiomic model and the immunotherapeutic outcomes of the patients.

**Result:**

For the immune cell infiltration radiomic prediction models in cohort 1, the AUC values achieved 0.765, 0.763, and 0.675 in the test set of CD3+ T, CD8+ T, and CD8+ TRM, respectively. While the AUC values for the TIME-immunotherapy predictive value were 0.651, 0.763, and 0.829 in the CD3-immunotherapy response model, CD8-immunotherapy response model, and CD8+ TRM-immunotherapy response model in cohort 2, respectively. The CD8+ TRM-immunotherapy model exhibited the highest predictive value and was significantly better than the CD3-immunotherapy model in predicting the immunotherapy response. The progression-free survival (PFS) analysis based on the predicted levels of CD3+ T, CD8+ T, and CD8+ TRM immune cell infiltration showed that the CD8+ T cell infiltration level was an independent factor (P=0.014, HR=0.218) with an AUC value of 0.938.

**Discussion:**

Our empirical evidence reveals that patients with substantial CD8+ T cell infiltration experience a markedly improved PFS compared with those with minimal infiltration, asserting the status of the CD8+ T cell as an independent prognosticator of PFS in the context of immunotherapy. Although CD8+ TRM cells demonstrated the greatest predictive accuracy for immunotherapy response, their predictive strength for PFS was marginally surpassed by that of CD8+ T cells. These insights advocate for the application of the proposed non-invasive radiomic model, which utilizes TIME analysis, as a reliable predictor for immunotherapy outcomes and PFS in NSCLC patients.

## Introduction

Lung cancer steadfastly remains the predominant cause of oncological mortality worldwide (1), with non-small cell lung cancer (NSCLC) accounting for an estimated 80–85% of these instances (2). With the emergence of immune checkpoint inhibitors (ICIs) as a treatment modality, immunotherapy has ascended to the forefront of therapeutic strategies for advanced lung cancer in recent years. Although clinical benefits are evident in approximately 20%–50% of patients (3, 4), there remains a substantial cohort that does not derive benefit from such therapies or may develop severe immune-related complications, including hyper-progressive disease manifestations, immune-related pneumonia, encephalitis, or even fatal outcomes post-treatment (5, 6). In light of these challenges, the expeditious and precise selection of patients who are anticipated to respond favorably to immunotherapy before its initiation has become an exigent priority.

The penetration and activation of immune cells within tumor sites underscore the fundamental mechanism behind the antitumor efficacy of ICIs (7). Precise evaluation of the tumor immune microenvironment (TIME) is instrumental in forecasting patient outcomes in response to ICI therapy (8–11). The extent of immune cell infiltration within a tumor is a critical factor in predicting the success of ICIs. Diverse immune cells contribute variably to the immune reaction and the immunotherapeutic pathway, highlighting the necessity of understanding the implications of various levels of immune cell infiltration on therapy outcomes. Research indicates a significant correlation between the density of CD3+ T cells (universal T-cell markers) and CD8+T cells (markers of cytotoxic T cells) within both the periphery and core of the tumor and patient prognosis (12–14). Furthermore, a specific subset of CD8+ T cells, known as tissue-resident memory CD8+ T cells (CD8+ TRM), characterized primarily by the elevated expression of the epithelial adhesion molecules CD103 and/or CD69, are permanently situated within mucosal epithelial tissues (15, 16). Evidently, CD8+ TRM cells are pivotal in tumor immune surveillance and the immunotherapy process (17, 18). Clinical research has demonstrated a positive correlation between the presence of tumor-infiltrating CD8+ tissue-resident memory T (TRM) cells and the prognosis for lung cancer patients (19, 20). TRM cells, vital elements of the early immune microenvironment, play a significant role in the recruitment of immune cells, with a high density of TRM cells being linked to both an improved prognosis and a favorable response to immunotherapy in NSCLC patients (18, 21). Thus, evaluating TIME across different dimensions, including CD3+ T, CD8+ T, and CD8+ TRM cells, affords a more nuanced insight into the immune landscape of tumors. Currently, the assessment of TIME is contingent upon the procurement of surgical or biopsy samples for the analysis of immune cells within tissues, a challenging endeavor for patients with advanced-stage NSCLC due to the impracticality of resection or the inherent heterogeneity of tumors. Consequently, the development of non-invasive techniques for the evaluation of TIME and the prediction of immunotherapeutic responses is of paramount importance.

Radiomics emerges as a cutting-edge methodology capable of converting computational medical imagery into analyzable data (22, 23). These medical images encapsulate macroscopic, cellular, and molecular insights into tumors, facilitating a deeper comprehension of tumor dynamics (22, 24). Owing to its superior performance in clinical diagnosis, prognostication, and therapeutic decision-making across a spectrum of cancers, when compared with conventional methods, radiomics has attracted burgeoning interest (25–28). It is posited as a pioneering approach for the non-invasive assessment of both the tumor and its immune microenvironment (29, 30). Furthermore, the correlation between imaging characteristics and the TIME has been extensively investigated, underscoring the significant potential of radiomic imaging biomarkers in evaluating tumor-infiltrating cells and forecasting the efficacy of immunotherapies (31–33). Recent research disclosed that radiomic features are predictive of NSCLC immunotherapy biomarkers, such as PD-L1 expression levels and tumor mutational burden (TMB) status (34). A comprehensive multicohort investigation revealed the utility of radiomic biomarkers in estimating CD8 cell counts and prognosticating clinical outcomes for patients undergoing immunotherapy (33).

In our endeavor, we have crafted an innovative immune cell infiltration radiomic prediction model designed to appraise the TIME of NSCLC and, additionally, to anticipate the outcomes of immunotherapy for patients afflicted with NSCLC. This non-invasive radiomic schema, grounded in the intricacies of TIME, exhibits enhanced predictive capabilities for determining patient responses to immunotherapy as well as progression-free survival (PFS) metrics among NSCLC sufferers. This model could potentially serve as an invaluable tool in the stratification of patients, aiding in the discernment of optimal candidates for immunotherapy.

## Materials and methods

### Study design and study population

This study was approved by the Institutional Research Review Board for human studies (Ethical Committee) of the Second Affiliated Hospital of Zhejiang University School of Medicine. Informed consent was waived as this was a retrospective study.

In clinical practice, patients requiring immunotherapy are often at an advanced stage at which surgical resection is no longer viable. Additionally, biopsy specimens may fail to provide a precise evaluation of the immune landscape due to their limited size and the tumor’s heterogeneity. To surmount this issue, data from patients who had surgical resections (cohort 1) were employed to construct a radiomic model capable of predicting the TIME. This model was then applied to forecast the TIME for patients under immunotherapy (cohort 2). Conclusively, the study delved into the association between the predicted TIME from the radiomic model and the immunotherapeutic outcomes of the patients.

**Figure 1:**
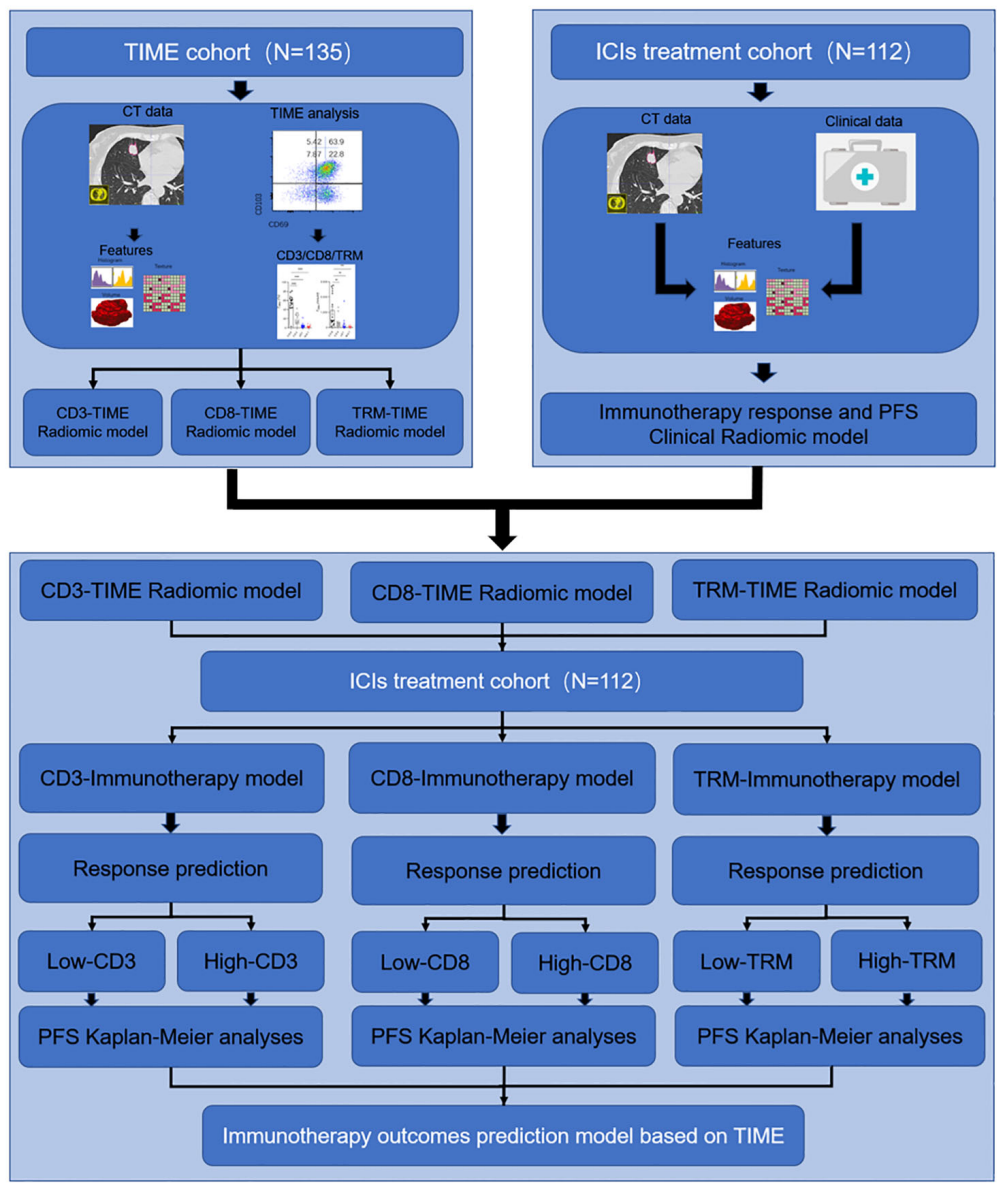
Study design for the immunotherapy response and PFS radiomic prediction model base on the TIME. In the first section, the TIME cohort was used for developing the immune cell infiltration prediction radiomic model of CD3+ T, CD8+ T, and TRM. In the second section, the ICI treatment cohort was used for developing the immunotherapy response clinical predicting model and PFS predicting model. In the third section, the TIME prediction model was used to predict the CD3+ T, CD8+ T, and TRM infiltration level and further predict the PFS in the ICI treatment cohort.

The overall study design is shown in **Figure 1**. This study retrospectively enrolled a TIME cohort (n = 135) and ICI treatment cohort (n = 112) from the Second Affiliated Hospital of Zhejiang University School of Medicine.

Within the ambit of the TIME cohort, we meticulously selected lung cancer cases diagnosed as primary NSCLC at stages IIB to IIIA over the period from 2016 to 2019. These patients had undergone lung tumor resections at the Department of Thoracic Surgery at the Second Affiliated Hospital of Zhejiang University School of Medicine. Inclusion in the analysis was contingent upon patients meeting the following prerequisites: 1) a confirmed diagnosis of NSCLC by a pathologist; 2) procurement of CT images from the picture archiving and communication system (PACS) within a month before surgery, with the stipulation that the lesions depicted must possess adequate image quality for subsequent radiomic analysis; 3) the availability of tissue samples deemed sufficient for the exploration of immune cell dynamics, coupled with comprehensive data on the infiltration ratios of CD3+ T cells, CD8+ T cells, and CD8+ TRM cells; and 4) the possession of a complete set of clinical records.

**Figure 2:**
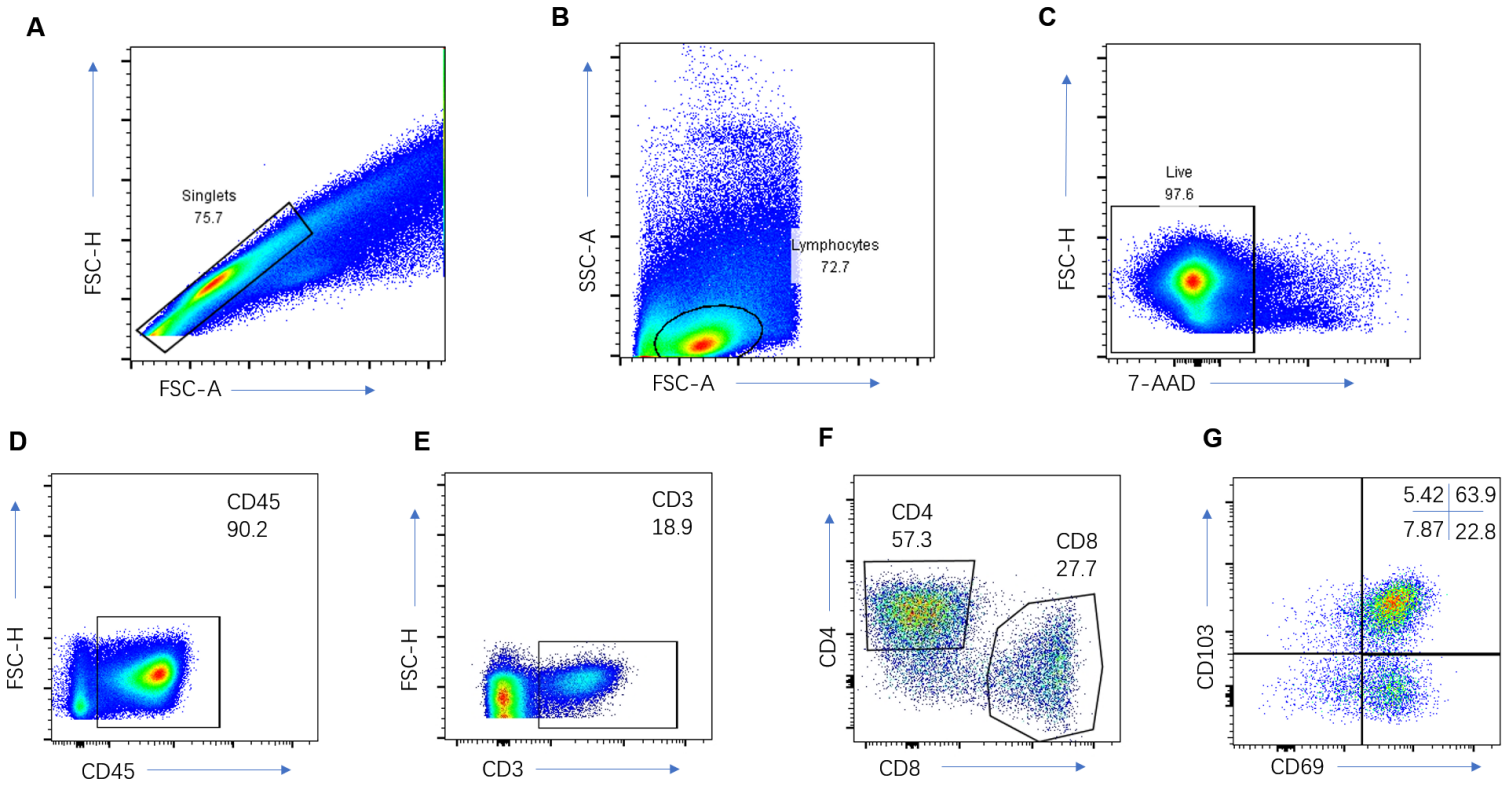
Immune cell analysis of the NSCLC. Representative gating strategy for the flow cytometric analysis of CD3+ T cells **(A–E)**, CD8+T cells **(A–F)**, and CD8+TRM cells **(A–G)**.

The analysis of immune cells encompassed three pivotal steps. 1) Immediately post-surgery, resected lung cancer tissue specimens were rapidly submerged in liquid nitrogen and thereafter preserved in a −80°C cryogenic freezer within the tissue repository. 2) For the preparation of a single-cell suspension of immune cells infiltrating lung cancer tissues, the specimens were first weighed and placed into a sterile container with Roswell Park Memorial Park (RPMI) 1640 medium supplemented with 5% fetal bovine serum, then swiftly transferred to be washed with PBS to eliminate necrotic tissue and lingering blood clots. Subsequently, tissues were meticulously diced into 1-mm^3 fragments using sterile ophthalmic scissors and subjected to a digestion protocol at 37°C for 2 h. The digestion mixture—enriched with RPMI 1640 medium, 10% fetal bovine serum, type I collagenase (1 mg/ml), type IV collagenase (1 mg/ml), and hyaluronidase (10 ng/ml)—was filtered through a 40 μm sieve. Post-centrifugation, cells were resuspended in the medium. 3) To ascertain the proportion of CD8+ TRMs within the infiltrating lymphocytes of lung cancer tissues, the single-cell suspension underwent a multicolor flow cytometry analysis (incorporating CD45, CD3, CD4, CD8, CD103, and CD69 markers) to precisely quantify the percentages of CD3+ T, CD8+ T, and CD8+ TRM cells present in the lung cancer tissues (**Figures 2A–G**). In the flow cytometry protocol, CD4 staining was employed to more accurately identify the subsets of CD8 cells and examine the distribution of TRM cells within the CD4 subsets. However, it is important to note that the current study did not further investigate the CD4 cells.

### Antibodies and flow cytometry

The antibodies used in this study were meticulously selected for their specificity and efficiency. These included CD45-APCCY7 (Clone #HI30, Cat#:304014, at a dilution of 1:100), CD3-BV421 (Clone #UCHT1, Cat#:300433, diluted at 1:100), CD8a-AF700 (Clone #RPA-T8, Cat#:301028, with a dilution ratio of 1:100), CD4-APC (Clone #RPA-T4, Cat#:300512, diluted at 1:100), CD103-FITC (Clone #Ber-ACT8, Cat#:350204, at a dilution of 1:100), CD69-PECY7 (Clone #FN50, Cat#:310911, diluted at 1:100), and 7-AAD (Cat#:420404, diluted at 1:200). These reagents were acquired from BioLegend.

To mitigate non-specific antibody binding, fresh tissue cells (at a concentration of 1×10^6/ml) were initially preincubated in a concoction comprising PBS, 2% fetal calf serum, and 0.1% (w/v) sodium azide, augmented with an FcγIII/IIR-specific antibody. Subsequently, these cells were labeled with various combinations of fluorochrome-conjugated antibodies for a duration of 15 min at ambient temperature. Following staining, cells were cleansed with PBS and sifted through a 70-μm mesh. A 7-AAD Viability Staining Solution was introduced 15 min before data acquisition to exclude dead cells, with data collection was carried out using a FACSCanto II system.

In the cohort receiving ICI treatment, we incorporated 112 patients diagnosed with stage IIIA to IVB NSCLC, all pathologically verified and administered ICIs, including toripalimab, nivolumab, and pembrolizumab, from 2018 to 2021. In clinical practice, to identify patients suitable for immunotherapy, we generally followed these screening procedures: prior to initiating immunotherapy, patients underwent a biopsy to confirm the pathological type and immunohistochemical testing to assess PD-L1 expression levels. Patients with high PD-L1 expression (>50%) were treated with immunotherapy alone, whereas those with low or unknown PD-L1 expression were considered for combined chemotherapy and immunotherapy. Some patients also had external genetic testing for TMB and other biomarkers. Patients were clinically staged to determine their suitability for surgery or neoadjuvant therapy. Immunotherapy or combined chemoradiotherapy was administered to patients who were not eligible for surgical resection or required preoperative adjuvant therapy. Patients with acute infections, interstitial lung disease, autoimmune diseases, or those on steroid therapy were excluded from the study. Patients included in our study were selected based on the fulfillment of the following specific criteria for the analysis: 1) pathological confirmation of NSCLC prior to the commencement of immunotherapy; 2) the acquisition of chest CT scans within a month preceding immunotherapy initiation, ensuring clarity and the absence of obfuscations such as pronounced respiratory motion or metallic artifacts (the images were processed using both lung and mediastinal windows, with slice thicknesses set at 1–2.5 mm for the lung window and 5 mm for the mediastinal window); 3) the presence of at least one measurable lesion within the patient; and 4) the completion of a chest CT follow-up post 1–2 cycles of immunotherapy.

To assess the efficacy of ICI treatment, patient evaluations were conducted in alignment with the immune response evaluation criteria in solid tumors (iRecist). Pre-treatment chest CT scans were meticulously reviewed to identify a measurable lesion, subsequently designated as the target lesion for measurement. Details such as the lesion’s location, initial dimensions, and pathological classification, as well as the specific immunotherapy regimen and dosage, were meticulously documented. Follow-up CT scans conducted after 1–2 cycles of immunotherapy were subsequently reviewed, with the target lesion’s response evaluated in accordance with iRecist guidelines. The PFS time was recorded for each patient, with the exception of those who underwent surgical resection.

### Medical image segmentation

For the purpose of this study, the Digital Imaging and Communications in Medicine (DICOM) data-sets of patients enrolled in the TIME cohort and ICI treatment cohort were retrieved from PACS. The CT scans were acquired using 16-slice or higher slice spiral GE healthcare, Siemens, and Philips CT systems. We used a 5 mm slice thickness standard reconstruction algorithm image and set the window level at 50 Hu and the window width at 2,000 Hu to ensure consistency throughout the segmentation procedure. The tumor outline in every slice was drawn as the region of interest (ROI) by a radiologist who had no knowledge of the tumors other than their locations by using a free open-source software package (ITK-SNAP, version 3.6.0; http://itksnap.org) to provide the ROI for computer-based image analysis.

### Feature extraction and matrix building

Radiomic feature extraction were carried out using a free open-source software package, FeAture Explorer software (FAE; V.0.5.5 https://github.com/salan668/FAE). The FAE is engineered to execute an automated exploration of assorted algorithmic combinations, subsequently appraising the efficacy of model constructions against the validation dataset (35). For each case, the Feature Extraction module of FAE facilitated the extraction of an extensive suite of 851 radiomic features from the CT imagery. These encompassed 162 first-order features, 14 morphological features, 216 gray-level co-occurrence matrix (GLCM) features, 144 gray-level run-length matrix (GLRLM) features, 144 gray-level size zone matrix (GLSZM) features, 45 neighborhood gray tone difference matrix (NGTDM) features, and 126 gray-level dependence matrix (GLDM) features. An enumerative catalog of these 851 features is presented in the **Supplementary Material**. To ascertain the intra-observer consistency, 20 cases were arbitrarily selected for the reiteration of ROI demarcation and feature extraction, and undertakings were performed by a second radiologist with 5 years of expertise in thoracic imaging diagnostics. The robustness of the radiomic features was assessed using intraclass correlation coefficients (ICC) to gauge the stability and reproducibility of the feature extraction process. An ICC value exceeding 0.80 was indicative of commendable reliability. Consequently, 730 features were deemed robust and compiled into a matrix for further feature selection.

Within the TIME cohort, the densities of infiltrated CD3+ T cells, CD8+ T cells, and CD8+ TRM cells were methodically ranked for 135 NSCLC patients. These populations were dichotomized into low-infiltrated and high-infiltrated groups based on the median value. The low-infiltrated group was encoded as 0, whereas the high-infiltrated group was encoded as 1. These encodings were integrated into the radiomic feature matrix, yielding separate matrices for CD3+ T, CD8+ T, and CD8+ TRM TIME radiomic features.

Regarding the ICI treatment cohort, all 112 NSCLC patients were classified based on their immunotherapy response: those with stable disease (SD) and progressive disease (PD) were categorized as having a poor immunotherapy response and encoded as 0; conversely, those with a complete response (CR) and partial response (PR) were categorized as having a favorable immunotherapy response and encoded as 1. These designations were also inserted into the radiomic feature matrix to formulate an immunotherapy response radiomic feature matrix. Follow-up data post-immunotherapy, notably the PFS, were meticulously recorded. Patients who experienced disease progression at the 12-month mark were marked as 1, whereas those without progression were marked as 0, allowing the formation of a PFS radiomic feature matrix.

### Radiomic model building

To construct the TIME prognostic model, the comprehensive matrices of CD3+ T, CD8+ T, and CD8+ TRM TIME radiomic features were inputted into the FAE software’s model exploration module. A computer-generated random algorithm partitioned the datasets, allocating 70% to the training set and 30% to the independent validation set. The dataset underwent standardization via Z-score normalization to mitigate variances and facilitate a more homogeneous comparison across the feature space, which was expansive. To streamline this feature space, we employed the Pearson correlation coefficient (PCC) to evaluate the pairwise similarity of features, excluding one feature from any pair that exhibited a PCC exceeding 0.99. Feature selection employed a multifaceted analytical approach: analysis of variance (ANOVA) and Kruskal–Wallis (KW) tests discerned features of statistical significance relative to the labels. In addition, recursive feature elimination (RFE) and relief methods were harnessed to identify and refine pertinent features in relation to the labels through recursive stratification and subset selection. Feature consideration was constrained to a range of 1 to 10. The evaluative strength of the features was tested through a battery of 10 machine learning algorithms. This diverse array included support vector machine (SVM), linear discriminant analysis (LDA), auto-encoder (AE), random forests (RF), linear regression (LR), logistic regression with least absolute shrinkage and selection operator (LASSO) (LRLasso), Ada-Boost (AB), decision tree (DT), Gaussian process (GP), and naive Bayes (NB). Model efficacy was ranked according to the area under the receiver operating characteristic curve (AUC) from the independent test set, with the optimal model for each CD3+ T, CD8+ T, and CD8+ TRM TIME radiomic prediction being selected on the basis of comparative AUC values. Parallel methodologies and parameters were applied in the ICI treatment cohort to develop immunotherapy response and PFS predictive models. These models were similarly validated, with the selection of the premier model informed by AUC statistics.

To predict immune cell infiltration in patients within the ICI treatment cohort, the retained features from the TIME and immunotherapy response models were used to craft the integrated TIME-immunotherapy response models. The predictive values, indicative of the infiltration levels of CD3+ T, CD8+ T, and CD8+ TRM, were extracted from these models. Subsequently, patients were categorized into low-infiltration and high-infiltration groups based on the derived prediction threshold values.

The prognostic implications of the infiltration levels of CD3+T, CD8+T, and CD8+TRM cells were gauged through Kaplan–Meier survival analysis. Additionally, multivariable Cox regression analysis was employed to determine the hazard ratios for specific T-cell infiltration levels, with adjustments made for concurrent infiltration levels of the other T-cell categories. This rigorous statistical approach provided a comprehensive assessment of the potential prognostic value of T-cell infiltration in patients receiving ICI therapy.

### Statistical analysis

Quantitative variables were delineated with precision as the mean accompanied by the standard deviation (M ± SD), whereas categorical data were succinctly expressed in terms of frequency and corresponding percentages where suitable. The chi-squared or Fisher’s exact test were judiciously applied to the categorical variables, whereas Student’s t-test or a Mann-Whitney U test were used for the continuous variables within the univariate analysis framework. The efficacy of the models was meticulously assessed by receiver operating characteristic (ROC) curves, with the model’s accuracy determined at the optimal cutoff point. This point was defined as the juncture where the sum of the model’s sensitivity and specificity reached its zenith, thereby maximizing the diagnostic potential. Additionally, the model’s discriminative capacity was quantitatively analyzed through the area under the ROC curve (AUC). The AUCs of different models were compared using Delong’s non-parametric method. Statistical analysis was performed using SPSS software (IBM SPSS Statistics Version 19.0, IBM Corp.), and the comparison of AUCs and the Kaplan–Meier analysis was carried out using MedCalc statistical software (version 22.023, MedCalc Software Ltd).

## Results

### Clinical characteristics

This study involved two cohorts of NSCLC patients from the Second Affiliated Hospital of Zhejiang University School of Medicine. The overarching design of the study is depicted in **Figure 1**. Within the TIME cohort, levels of infiltration by CD3+ T, CD8+ T, and CD8+ TRM immune cells were meticulously recorded, alongside chest CT scan data, to facilitate the establishment of the TIME radiomic model. The cohort consisted of 135 patients staged IB to IIIA, all confirmed by pathology following surgery. The demographic breakdown included 63 males and 72 females, with a median age of 61.5 years (range: 34–83 years) and a standard deviation of ±9.1. Histopathological evaluations revealed 113 cases of adenocarcinoma, 18 of squamous carcinoma, and 4 of other cancer types.

In the ICI treatment cohort, 112 patients at stages IIIA to IVB were surveyed, consisting of 90 males and 22 females, with a median age of 64.4 years (range: 45–83 years) and a standard deviation of ±8.1. Treatment modalities included monotherapy with ICIs in 8 cases, chemotherapy in combination with immunotherapy in 102 cases, and immunotherapy following unsuccessful tyrosine kinase inhibitor (TKI) therapy in 2 cases. Post-immunotherapy, 9 out of the 112 patients underwent surgical resection. Regarding histopathology, there were 47 adenocarcinoma cases, 58 squamous carcinoma cases, and 7 cases of other types. The efficacy of immunotherapy was classified as CR in 8 patients, PR in 76 patients, SD in 18 patients, and PD in 10 patients, with detailed breakdowns provided in **Table 1**.

**Table 1:** Characteristics of the NSCLC patients in each cohort.

	TIME cohort	ICIs cohort
Age (years)		
Median	61.5 ± 9.1	64.4 ± 8.1
Gender		
Male	63 (46.7%)	90 (80.4%)
Female	72 (53.3%)	22 (19.6%)
Smoking status		
Smoker	37 (27.4%)	42 (37.5%)
Non-smoker	98 (72.6%)	70 (62.5%)
Pathological type		
Adenocarcinoma	113 (83.7%)	47 (42.0%)
Squamous carcinoma	18 (13.3%)	58 (51.8%)
Others	4 (3%)	7(6.3%)
Clinical stage		
Ib	91 (67.4%)	
IIa	8 (5.9%)	
IIb	21 (21%)	
IIIa	15 (11.1)	12 (10.7%)
IIIb		39 (34.8%)
IIIc		32 (28.6%)
IVa		29 (25.9%)
ICI treatment response		
CR		8 (7.1%)
PR		76 (67.9%)
SD		18 (16.1%)
PD		10 (8.9%)
Progression-free survival		
Excluded cases		9 (8.0%)
Censored cases		2 (1.8%)
Median (months)		10.3 ± 9.5

### Predictive value of the radiomic model for the TIME

**Figure 3:**
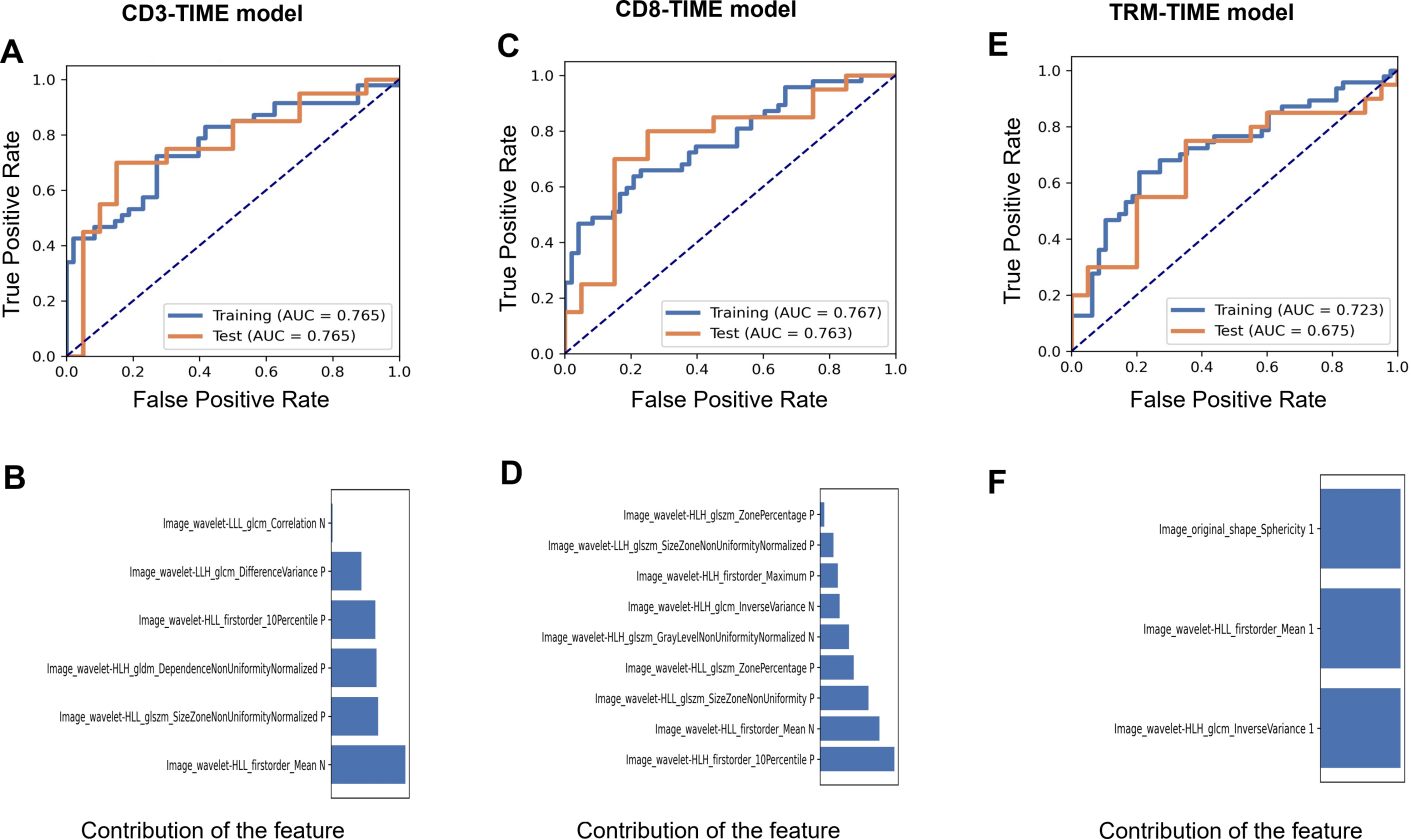
The performance of the CD3, CD8, and TRM TIME prediction models. **(A)** AUCs of the receiver operating characteristic (ROC) curves of the training and test sets in the CD3-TIME prediction model. **(B)** The contribution of retained features in the model **(B)**. **(C)** AUCs and the contribution of retained features **(D)** in the CD8-TIME prediction model. **(E, F)** AUCs **(E)** and the contribution of retained features **(F)** in the TRM-TIME prediction model.

Radiomic prediction models were established for CD3+ T, CD8+ T, and CD8+ TRM immune cell infiltration levels, named CD3-TIME, CD8-TIME, and TRM-TIME models, respectively. The CD3-TIME model retained six radiomic features and achieved AUC values of 0.765 [0.67–0.86; 95% confidence interval (CI)] and 0.765 (0.61–0.92; 95% CI) for the training and test sets, respectively (**Figure 3A**). Analysis using FAE software indicated that a six-feature model demonstrated the best performance, as shown in **Figure 3B**. The feature selection ANOVA and the LR classifier exhibited the most favorable performance (**Supplementary Figures S1A, B**). Within the CD8-TIME model, nine radiomic features were retained, resulting in AUC values of 0.767 (0.67–0.86; 95% CI) and 0.763 (0.61–0.92; 95% CI) for the training and test sets, respectively (**Figure 3C**). The contribution of these features to the model is illustrated in **Figure 3D**. **Supplementary Figures S2A, B** presents the performance of the feature selection and classifier. Regarding the TRM-TIME model, three radiomic features were retained, with AUC values of 0.723 (0.63–0.83; 95% CI) and 0.675 (0.50–0.85; 95% CI) for the training and test sets, respectively (**Figure 3E**). The contribution of these features is displayed in **Figure 3F**, and **Supplementary Figures S3A, B** provides the feature selection and classifier performance. The retained radiomic features for each model are presented in **Table 2**. To compare the performance of the models, Delong’s test was employed for pairwise comparison of ROC curves. The results showed no significant differences among the CD3+ T, CD8+ T, and TRM TIME prediction models (CD3 vs. CD8, *P*=0.9646; CD3 vs. TRM, *P*=0.4370; CD8 vs. TRM, *P*=0.4652).

**Table 2:** The retained radiomics features in the TIME prediction model.

	Reserved features
CD3_TIME model	Image_wavelet-HLL_firstorder_10Percentile Image_wavelet-LLH_glcm_DifferenceVariance Image_wavelet-HLH_gldm_DependenceNonUniformityNormalized Image_wavelet-HLL_firstorder_Mean Image_wavelet-LLL_glcm_Correlation Image_wavelet-HLL_glszm_SizeZoneNonUniformityNormalized
CD8_TIME model	Image_wavelet-HLH_glcm_InverseVariance Image_wavelet-HLH_glszm_GrayLevelNonUniformityNormalized Image_wavelet-HLH_firstorder_10Percentile Image_wavelet-HLL_glszm_ZonePercentage Image_wavelet-LLH_glszm_SizeZoneNonUniformityNormalized Image_wavelet-HLH_firstorder_Maximum Image_wavelet-HLH_glszm_ZonePercentage Image_wavelet-HLL_glszm_SizeZoneNonUniformity Image_wavelet-HLL_firstorder_Mean
TRM_TIME model	Image_wavelet-HLH_glcm_InverseVariance Image_wavelet-HLL_firstorder_Mean Image_original_shape_Sphericity

### Immunotherapy outcome radiomic predictive model based on clinical data

**Figure 4:**
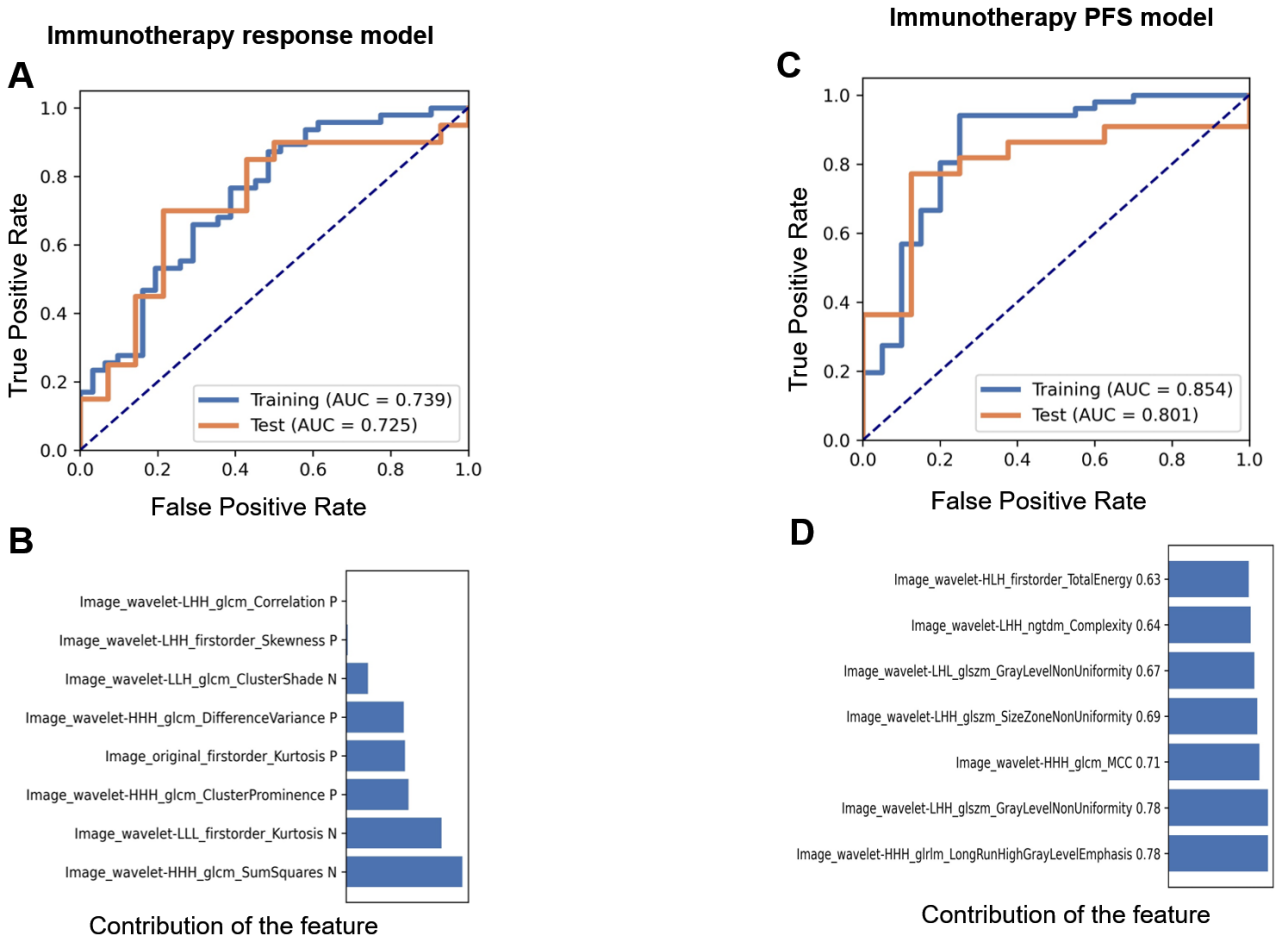
The performance of the immunotherapy response and PFS models. AUCs of the receiver operating characteristic (ROC) curves of the training and test sets in the immunotherapy prediction model **(A)** and the contribution of retained features **(B)** based on clinical data. AUCs **(C)** and the contribution of retained features **(D)** in the PFS prediction model based on clinical data.

In the ICI treatment cohort, we developed an immunotherapy response prediction model based on the immune response evaluation criteria in solid tumors (iRECIST) using clinical follow-up data after immunotherapy. The cohort consisted of 112 patients, who were randomly divided into a training set (78 cases) and a test set (34 cases). The resulting model was referred to as the immunotherapy response model. The AUC values for the training and test sets were 0.739 (0.62–0.85; 95% CI) and 0.725 (0.54–0.91; 95% CI), respectively (**Figure 4A**). The model retained eight features, and their contributions are presented in **Figure 4B**. Relief in feature selection algorithms and LR in classifiers demonstrated a superior performance to other methods (**Supplementary Figures S4A, B**). Additionally, when using the patients’ 12-month PFS as a label to establish a prediction model, the final model retained nine features. The AUC values for the training and test sets were 0.854 (0.74–0.97; 95% CI) and 0.801 (0.62–0.98; 95% CI), respectively (**Figure 4C**). The contribution of these features is shown in **Figure 4D**. Relief in feature selection algorithms and GP in classifiers exhibited better performance than other methods (**Supplementary Figures S5A, B**). **Table 3** displays the retained radiomic features for each model.

**Table 3:** The retained radiomics features in the response and PFS prediction models.

	Reserved features
Response model	Image_wavelet-LLL_firstorder_Kurtosis Image_original_firstorder_Kurtosis Image_wavelet-LHH_firstorder_Skewness Image_wavelet-LHH_glcm_Correlation Image_wavelet-LLH_glcm_ClusterShade Image_wavelet-HHH_glcm_ClusterProminence Image_wavelet-HHH_glcm_DifferenceVariance Image_wavelet-HHH_glcm_SumSquares
PFS model	Image_wavelet-HHH_glrlm_LongRunHighGrayLevelEmphasis Image_wavelet-LHH_glszm_GrayLevelNonUniformity Image_wavelet-HHH_glcm_MCC Image_wavelet-LHH_glszm_SizeZoneNonUniformity Image_wavelet-HLH_firstorder_Skewness Image_wavelet-LHL_glszm_GrayLevelNonUniformity Image_wavelet-LHH_ngtdm_Complexity

### The predictive value of TIME radiomic features for the immunotherapy response

**Figure 5:**
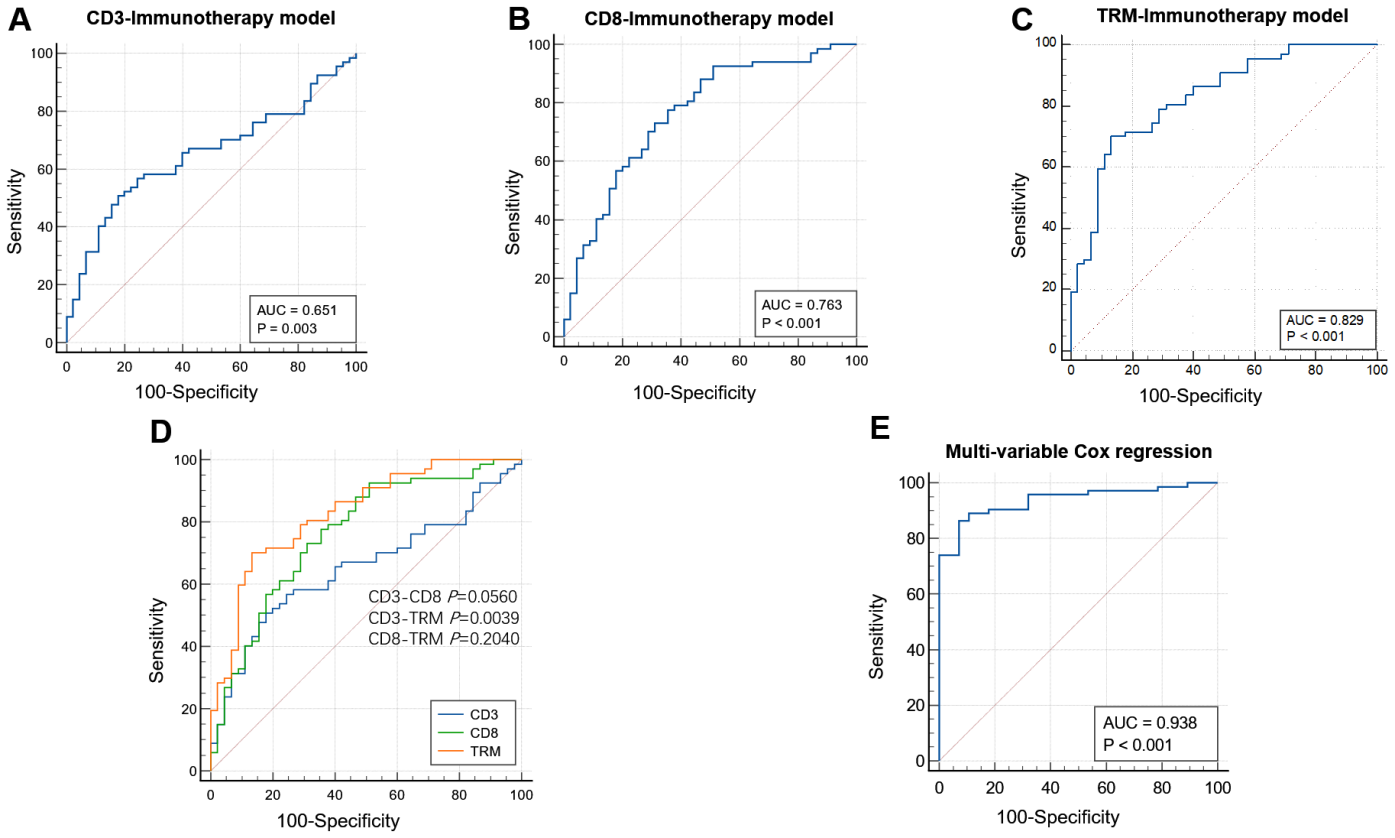
The performance of immunotherapy response models based on TIME. AUCs of the receiver operating characteristic curves of the CD3 **(A)**, CD8 **(B)**, and TRM **(C)** immunotherapy response models based on CD3, CD8, and TRM TIME radiomic features. Comparison among the three models. The TRM immunotherapy model had the highest predictive value and was better than the CD3 immunotherapy model (0.829 vs. 0.651, *P*=0.0039) in predicting the immunotherapy response. There were no significance in the CD3-CD8 immunotherapy model (0.651 VS. 0.763 P=0.0560) and CD8-TRM immunotherapy model (0.763 vs. 0.829, *P*=0.2040) **(D)**. The AUC value was 0.938 after adjusting the CD3, CD8, and TRM infiltration level according to multivariable Cox regression analysis **(E)**.

Using the radiomic features retained in the TIME model and immunotherapy response model, we constructed the TIME-immunotherapy response model. This model provided predictive values for the infiltration levels of CD3+ T, CD8+ T, and CD8+ TRM immune cells in each patient. The AUC values for the TIME-immunotherapy predictive value were 0.651 (0.56–0.74; 95% CI), 0.763 (0.67–0.84; 95% CI), and 0.829 (0.75–0.89; 95% CI) in the CD3-immunotherapy response model (**Figure 5A**), CD8-immunotherapy response model (**Figure 5B**), and TRM-immunotherapy response model (**Figure 5C**), respectively. To compare the performance of these models, Delong’s test was conducted for pairwise comparison of the ROC curves (**Figure 5D**). The TRM-immunotherapy model exhibited the highest predictive value and was significantly better than the CD3-immunotherapy model (0.829 vs. 0.651, P=0.0039) in predicting the immunotherapy response. However, there was no significant difference between the CD3-CD8 immunotherapy model (0.651 vs. 0.763, *P*=0.0560) or the CD8-TRM immunotherapy model (0.763 vs. 0.829, *P*=0.2040).

### The relationship between the immune cell infiltration level and PFS

**Figure 6:**
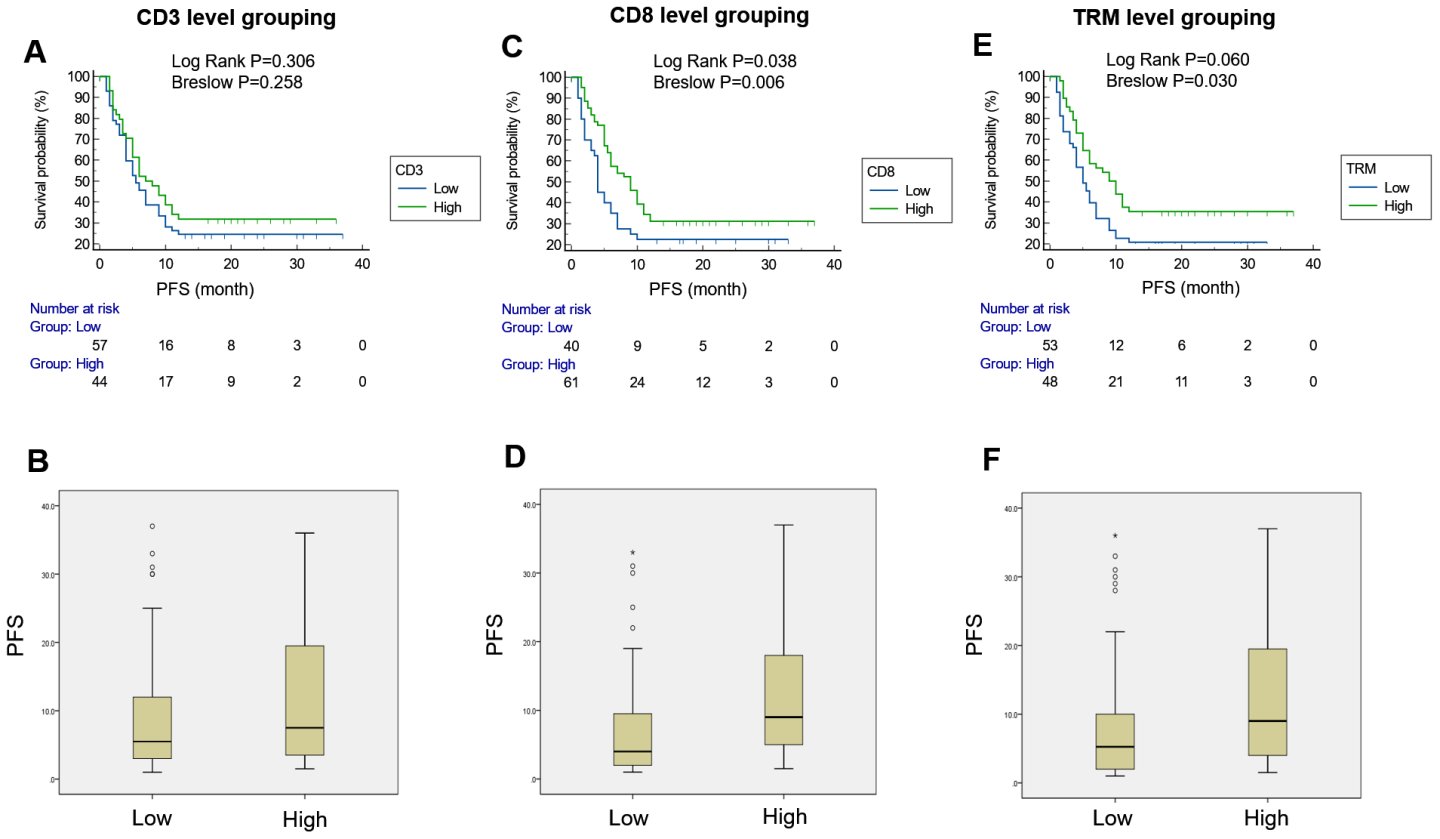
PFS survival curves for the CD3, CD8, and TRM infiltration level grouping. There was no significant difference in PFS between the two groups [low-CD3 vs. high-CD3, 12.75 ± 1.89 vs. 15.17 ± 2.18; log-rank test, *P*=0.306; Breslow test, *P*=0.258] **(A, B)**, indicating that the level of CD3+T cell infiltration was not predictive of PFS. There was a significant difference in PFS between the two groups (low-CD8 vs. high-CD8, 10.36 ± 1.96 vs. 15.70 ± 1.87; log-rank test, *P*=0.038; Breslow test, *P*=0.006) **(C, D)**, indicating that the level of CD8+T cell infiltration was effective in predicting PFS. There was a significant difference in PFS between the two groups (low-TRM vs. high-TRM, 11.51 ± 1.82 VS. 16.48 ± 2.19; log-rank test, *P*=0.060; Breslow test, *P*=0.030) **(E, F)**, indicating that the CD8+ TRM cell infiltration level was effective in predicting PFS in the early period of immunotherapy.

In the ICI treatment cohort, patients who received monotherapy and surgical resection were excluded from the analysis because the survival time of neoadjuvant therapy patients differs from those receiving combined chemotherapy and immunotherapy. Consequently, 101 cases were included for further analysis. The level of immune cell infiltration predicted by the TIME-immunotherapy model was used to divide the patients into different groups: a low-CD3+ T cell infiltration group and high-CD3+T cell infiltration group, a low-CD8+T cell infiltration group and high-CD8+T cell infiltration group, and a low-TRM cell infiltration group and high-TRM cell infiltration group. Kaplan–Meier analyses were then performed to assess the PFS based on the predicted levels of CD3+ T, CD8+ T, and TRM immune cell infiltration. For the CD3+ T cell infiltration level grouping, the disease control rate (DCR) for the low-CD3+ T cell infiltration and high-CD3+ T cell infiltration groups was 24.6% (14 in 57 cases) vs. 31.8% (14 in 44 cases) (*P*=0.491), and the mean progress time for the low-CD3+ T cell infiltration and high-CD3+ T cell infiltration groups was 12.75 ± 1.89 vs. 15.17 ± 2.18 months (log-rank *P*=0.306; Breslow *P*=0.258), with no significant difference between the two groups (**Figures 6A, B**). Regarding the CD8+ T cell infiltration level grouping, the DCR for the low-CD8+ T cell infiltration and high-CD8+ T cell infiltration groups was 22.5% (9 in 40 cases) vs. 31.1% (19 in 61 cases) (*P*=0.342), and the mean progress time for the low-CD8+ T cell infiltration group and high-CD8+ T cell infiltration group was 10.36 ± 1.96 vs. 15.70 ± 1.87 months (log-rank *P*=0.038; Breslow *P*=0.006), showing a significant difference between the two groups (**Figures 6C, D**). In the case of the CD8+ TRM cell infiltration level grouping, the DCR for the low-TRM cell infiltration group and high-TRM cell infiltration groups was 22.2% (12 in 54 cases) vs. 34.0% (16 in 47 cases) (*P*=0.186), and the mean progress time in the low-TRM cell infiltration group and high-TRM cell infiltration group was 11.51 ± 1.82 vs. 16.48 ± 2.19 months (log-rank *P*=0.060; Breslow *P*=0.030). There was a significant difference between the two groups in the early stage of treatment, but no significance in the later stage (**Figures 6E, F**). To determine the independent factors predicting immunotherapy patients progressing at 12 months, a multivariable Cox regression analysis was performed by adjusting for CD3, CD8, and TRM infiltration levels. The results showed that the CD8+ T cell infiltration level was an independent factor (*P*=0.014, HR=0.218) with an AUC value of 0.938 (0.893–0.983; 95% CI) (**Figure 5E**).

## Discussion

Immunotherapy has substantially changed the therapeutic strategies for lung cancer patients (36). Unfortunately, only 20–50% of patients with advanced solid tumors respond positively to immunotherapy treatment (4), and some patients even have adverse effects or fatal complications after treatment (5, 6). Predicting patients who are most likely to respond to immunotherapy will provide helpful information for treatment strategy (10, 11). Several studies have shown that pre-existing tumoral and peritumoral immune infiltration correlates with patient responses to anti-programmed cell death protein (PD)-1 and anti-programmed cell death ligand 1 (PD-L1) immunotherapy (11, 37, 38). With the increasing use of immunotherapy in cancer, knowledge of an individual’s immune status could help identify those who will respond positively to treatment (10, 11). In our study, we developed a TIME-based radiomic model to predict the ICI treatment response and PFS. This study established radiomic prediction models for CD3+ T, CD8+ T, and CD8+ TRM immune cell infiltration levels, and then predicted the level of immune cell infiltration in tumor tissues of NSCLC patients in the immunotherapy cohort based on the radiomic model of the TIME. Finally, we investigated the effects of CD3+ T, CD8+ T, and CD8+ TRM immune cell infiltration levels on patients’ immunotherapy response and PFS (15–17, 39, 40).

Retrospective analyses of patient populations treated with ICIs have revealed that there are classes of TIMEs that are associated with those tumors that are more prone to ICI responsiveness (11, 37, 38). Deeper analysis of the complexity within the TIME is likely to reveal and identify patient populations that will respond to ICI therapy. T cells are known to be a key group of anti-tumor immune cells. Previous studies have shown that CD3+ T cells and CD8+ T cells at the tumor margin and within the tumor are closely associated with prognosis. Patients with T cells present within the tumor benefit preferentially from PD-1 therapy (10). Therefore, the infiltration density of T cells in the tumor microenvironment and the key antitumor immune cells such as CD8+ T cells will be powerful in predicting the response to immunotherapy (39). A study that evaluated different variables associated with immunotherapy response across different tumor types found that, among 36 variables, CD8+ T cell abundance was the most predictive of the response to immunotherapy across cancer types, followed by the TMB and the fraction of samples with high *PD-1* gene expression (39). In addition, tissue-resident memory CD8+ T (CD8+ TRM) cells, which are characterized by the high expression of the epithelial adhesion molecules CD103 and/or CD69, are a key subpopulation of CD8+ T cells (15, 16). Studies have shown that CD8+ TRM cells play a key role in tumor immunosurveillance and immunotherapy (17, 40).

However, a practical limitation for the histological characterization of T lymphocyte infiltration in clinical practice is the scarce availability of tumor tissue. In the majority of cases, NSCLC was diagnosed at advanced stages of disease, when radical resection is not feasible and the tumor tissue is limited or heterogeneous from small biopsy samples (41). It is necessary to explore non-invasive and efficient methods to assess the immune microenvironment.

Radiomics involves the analysis and translation of medical images into quantitative data (30, 42). High-dimensional imaging data allow an in-depth characterization of tumor phenotypes, with the underlying hypothesis that imaging reflects not only macroscopic but also cellular and molecular properties of tissues. The objective of radiomics is to generate image-driven biomarkers that serve as instruments that provide a deeper understanding of cancer biology to better aid clinical decisions (43). Radiomic prediction models have been widely applied in the diagnoses, prognosis, and treatment response prediction of tumors.

In our study, we developed a radiomic prediction model based on an analysis of immune cells using flow cytometry in NSCLC resection samples to predict the TIME. The AUC values for the testing set in the CD3+ T, CD8+ T, and CD8+ TRM TIME prediction models were 0.765, 0.763, and 0.675, respectively (**Figure 3**). These results demonstrate the predictive power of our radiomic model in determining the infiltration levels of immune cells in the tumor microenvironment. Other studies have also investigated the use of radiomic models to predict the immune phenotype of tumors. For example, a study developed a radiomic signature for CD8 cells and validated it in multiple cohorts, showing a promising predictive ability for immune phenotypes. Additionally, this study demonstrated that the radiomic signature could infer clinical outcomes in an ICI treatment cohort (33). However, it is important to note that the dataset used in this study included various cancer types, which may limit the accuracy and specificity of the radiomic model when applied specifically to lung cancer patients.

Another study used radiomics to study the TIME in NSCLC patients and classified the TIME into “hot” and “cold” types based on the expression of PD-L1 and the number of tumor-infiltrating lymphocytes. Radiomic features could accurately discriminate between hot and cold tumors, and these groups exhibited different overall survival and disease-free survival rates. Validation in an additional cohort further confirmed the prognostic impact of the radiomic signature (44).

In our study, we constructed a radiomic model to predict the infiltrations levels of CD3+ T, CD8+ T, and CD8+ TRM immune cells in the TIME. The AUC values for predicting ICI treatment response were 0.651 for CD3+ T, 0.763 for CD8+ T, and 0.829 for CD8+ TRM, indicating that CD8+ TRM infiltration level had the highest predictive performance. Survival analysis revealed that there was no significant difference in PFS between patients with low and high CD3+ T cell infiltration levels. However, patients with high levels of CD8+ T cell infiltration had a significantly better PFS than those with low levels. Multivariable Cox regression analysis showed that the CD8+ T cell infiltration level was an independent predictor of immunotherapy patients progressing at 12 months, with an AUC of 0.938. On the other hand, high levels of CD8+ TRM cell infiltration only predicted better PFS in the early stage of immunotherapy, suggesting that CD8+ T cell infiltration had a stronger predictive ability for PFS in the later stages of immunotherapy. Overall, our study demonstrates the utility of the radiomic model in predicting immune cell infiltration levels and the response to immunotherapy in NSCLC patients, with CD8+ T cell infiltration showing the strongest association with PFS.

T cells, co-expressing CD3, and their subset, cytotoxic T cells, which express CD8, are pivotal in the TIME. The presence of CD3+ T cells serves as a surrogate for T-cell abundance, and CD8+ T cells indicate cytotoxic activity. Our study demonstrates that, in NSCLC patients, CD3+ T cell levels do not consistently predict ICI treatment outcomes or PFS. Conversely, CD8+ T cell infiltration is a reliable predictor for both ICI response and PFS. Surprisingly, CD8+ TRM cells, a CD103+ subset of CD8+ T cells within epithelial tissues, exhibit a slightly diminished role in antitumor immunity compared with CD8+ T cells. This finding contrasts with the prevailing literature and our initial hypotheses. Despite their potent recruitment by CD103 and their established correlation with a favorable prognosis (19, 20, 45), CD8+ TRM cells’ predictive capacity for PFS post-ICI treatment is not as robust as that of CD8+ T cells, particularly in the later treatment stages. The multivariate analyses reveal that TRM cells are an independent predictive factor for clinical outcomes in lung cancer—emphasizing their significance at the apex of the local immune response, in which TRM concentrations surpass those of effector CD8+ T cells by tenfold and persist beyond 30 days when effector cells are no longer detectable (46). Theoretically, TRM cells should be superior in antitumor efficacy; however, the discrepancies observed might be attributable to our study’s small sample size and the heterogeneity in clinical stages and treatment regimens.

The study’s retrospective single-center design is a limitation, compounded by the varying stages of NSCLC patients and the absence of direct immune cell analysis in the immunotherapy cohort. The data used in the TIME cohort were obtained from a previous study that aimed to explore the immune regulatory mechanisms in cancer. We utilized the radiomics model in the TIME cohort to predict the immune cell infiltration level, which does not involve a direct comparison between the two cohorts. We consider that the different stages of cancer in the cohorts do not interfere with the performance of the radiomics model in predicting immune cell infiltration. When we analyzed the DCR between the high and low T-cell infiltrations, there was no significant difference between the CD3+, CD8+, and TRM groups; we suppose that the radiomic model can only approximately predict the levels of immune cell infiltration. Additionally, our sample size was quite limited, which may have been the reason for the lack of statistical significance in the DCR. The distribution of squamous cell carcinoma patients in the TIME and ICI cohorts can be attributed to a few factors. In the TIME cohort, all patients underwent tumor resection procedures for earlier clinical stages. In clinical practice, patients in these early stages are more likely to have adenocarcinoma. On the other hand, the ICI cohort consisted of patients in advanced stages who received immunotherapy. In advanced stages, the number of squamous cell carcinoma patients tends to be higher. This may be due to different patterns of disease progression and treatment response in squamous cell carcinoma compared with adenocarcinoma. Furthermore, we observed that patients with squamous cell carcinoma respond more positively to immunotherapy than those with adenocarcinoma. Consequently, a higher proportion of squamous cell carcinoma cases were included in immunotherapy trials in clinical practice. These factors necessitate prospective multicenter studies to increase the predictive accuracy of radiomic models.

In conclusion, our research supports the use of radiomic imaging biomarkers in non-invasively forecasting the TIME and, by extension, the response to immunotherapy and PFS in NSCLC patients. Importantly, it underscores the superior predictive role of CD8+ T cells over CD3+ T cell infiltration levels, which could inform preclinical immunotherapeutic strategies for NSCLC.

## Data Availability

The original contributions presented in the study are included in the article/**Supplementary Material**. Further inquiries can be directed to the corresponding authors.

## Supplementary material

Supplementary Figure 1 The performance of feature selection and classifier in the CD3-TIME prediction model. The ANOVA algorithm in the feature selection **(A)** and LR in the classifier **(B)** had the best performance.

Supplementary Figure 2 The performance of feature selection and classifier in the CD8-TIME prediction model. The ANOVA algorithm in the feature selection **(A)** and LDA in the classifier **(B)** had the best performance.

Supplementary Figure 3 The performance of feature selection and classifier in the TRM-TIME prediction model. The RFE algorithm in the feature selection **(A)** and AE in the classifier **(B)** had the best performance.

Supplementary Figure 4 The performance of feature selection and the classifier in the immunotherapy response prediction model. The relief algorithm in the feature selection **(A)** and LR in the classifier **(B)** had the best performance.

Supplementary Figure 5 The performance of feature selection and the classifier in the PFS prediction model. The relief algorithm in the feature selection **(A)** and GP in the classifier **(B)** had the best performance.

Supplementary Figure 6 The FAE panel. The main panel **(A)**, feature preprocessing window **(B)**, model exploration window **(C)**, and result visualization window **(D)** of FeAture Explorer V.0.5.5.
