# Supplementary figures and images for: The NSCLC immunotherapy response predicted by tumor-infiltrating T cells via a non-invasive radiomic approach
Source: Front Immunol. 2024 Sep 9;15:1379812. doi: 10.3389/fimmu.2024.1379812 (PMC11416977; doi:10.3389/fimmu.2024.1379812)

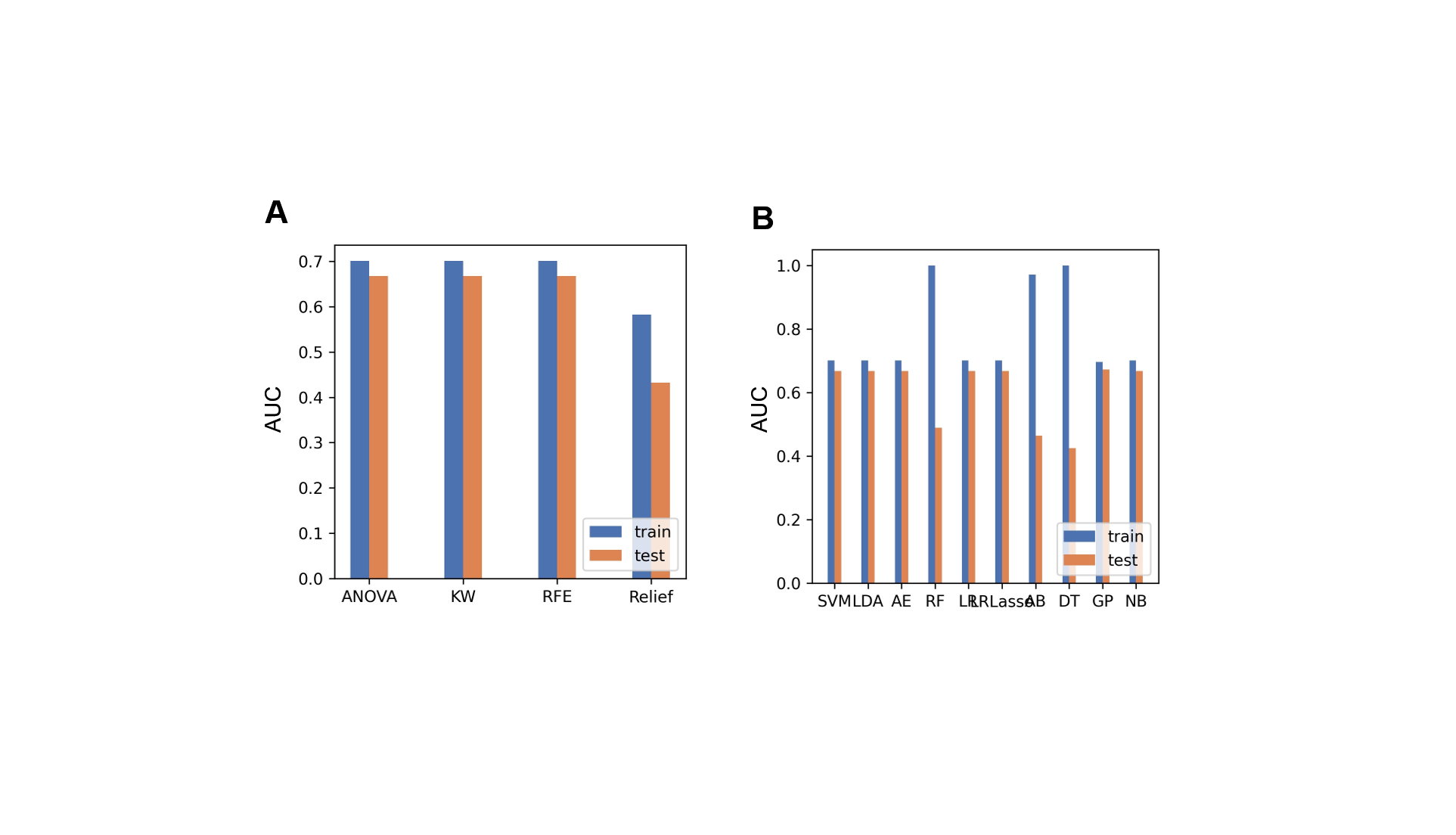

Supplement: Supplementary Figure 1 — The performance of feature selection and classifier in the CD3-TIME prediction model. The ANOVA algorithm in the feature selection (A) and LR in the classifier (B) had the best performance. [file Image1.tif]

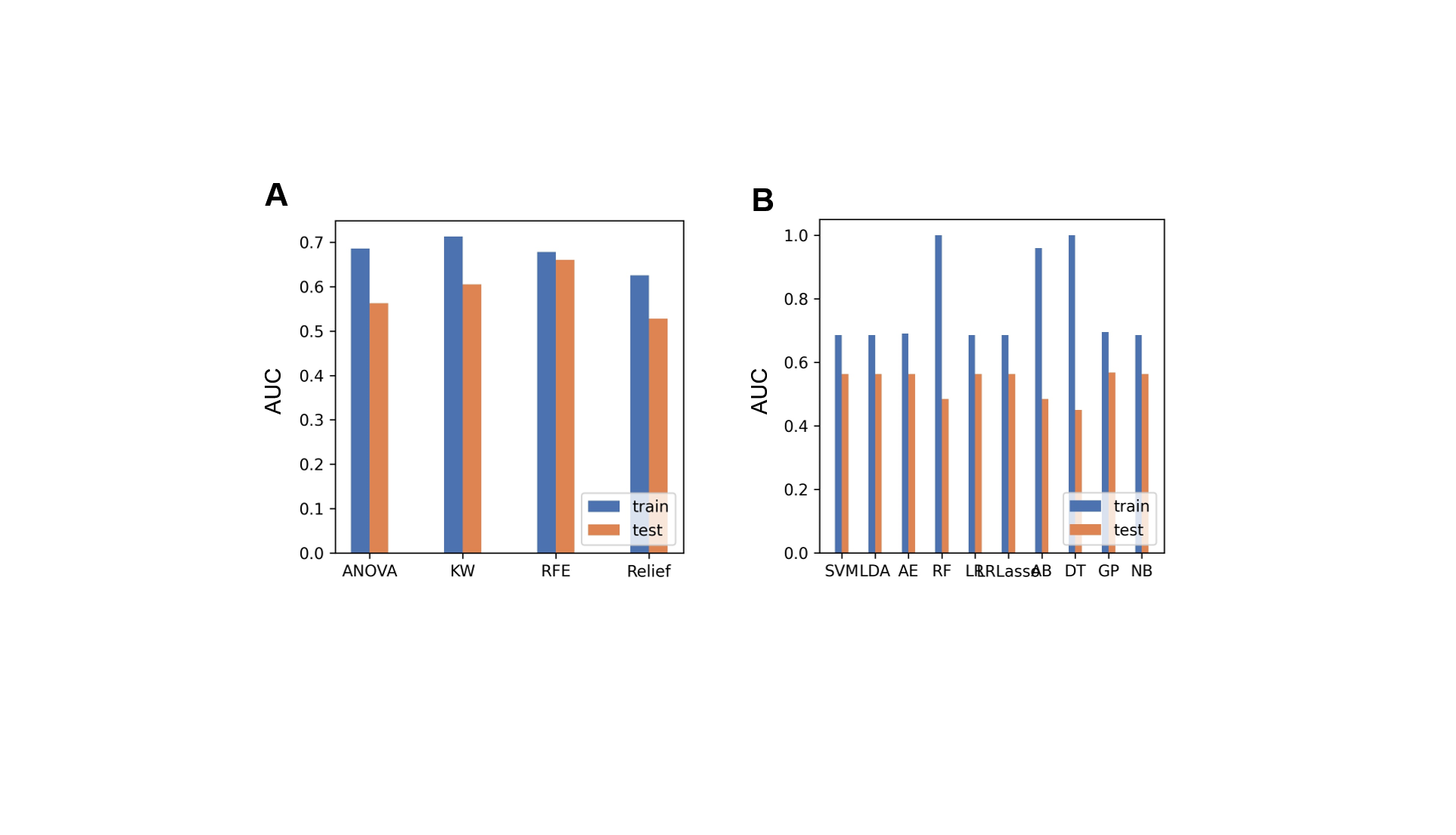

Supplement: Supplementary Figure 2 — The performance of feature selection and classifier in the CD8-TIME prediction model. The ANOVA algorithm in the feature selection (A) and LDA in the classifier (B) had the best performance. [file Image2.tif]

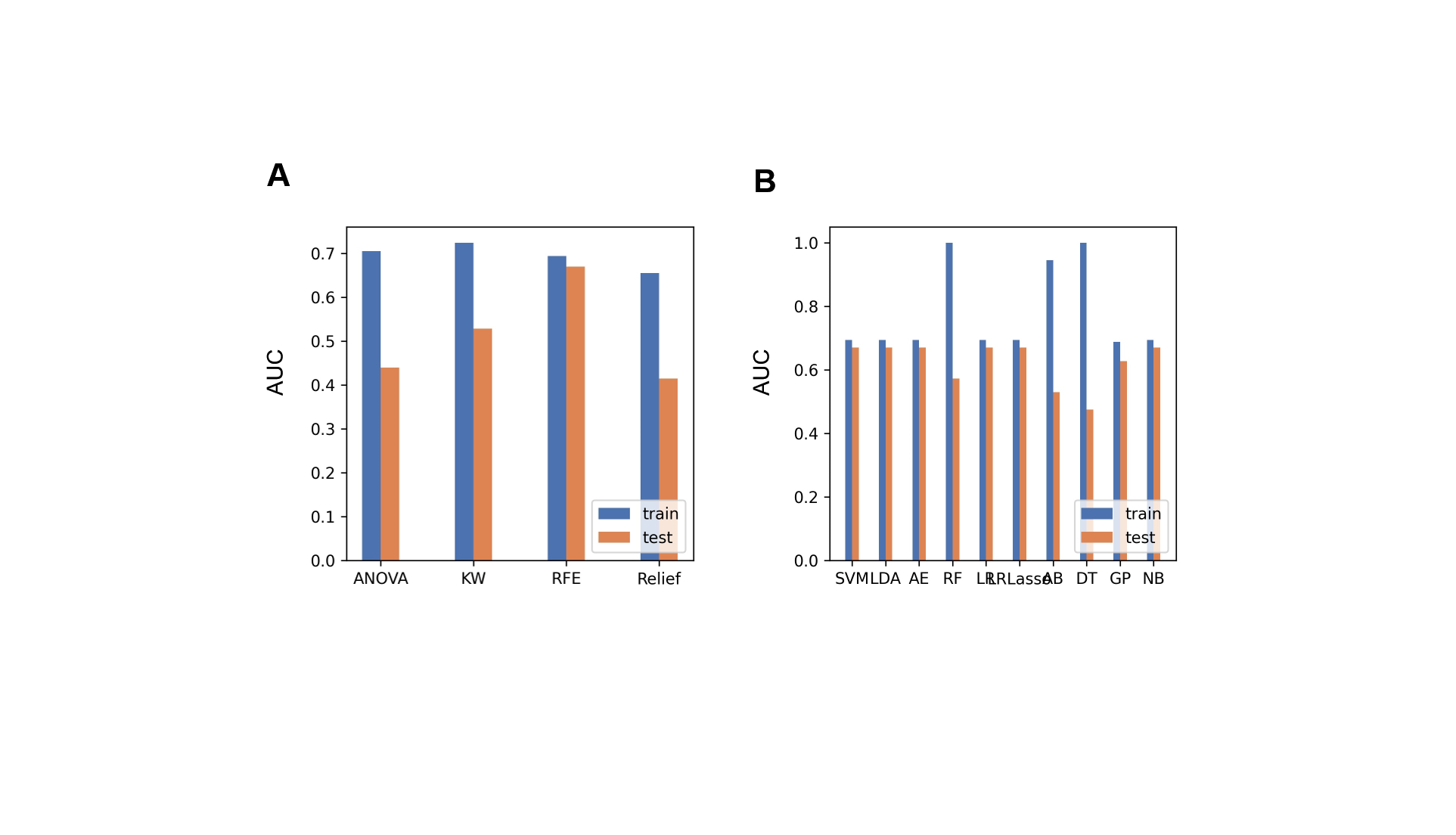

Supplement: Supplementary Figure 3 — The performance of feature selection and classifier in the TRM-TIME prediction model. The RFE algorithm in the feature selection (A) and AE in the classifier (B) had the best performance. [file Image3.tif]

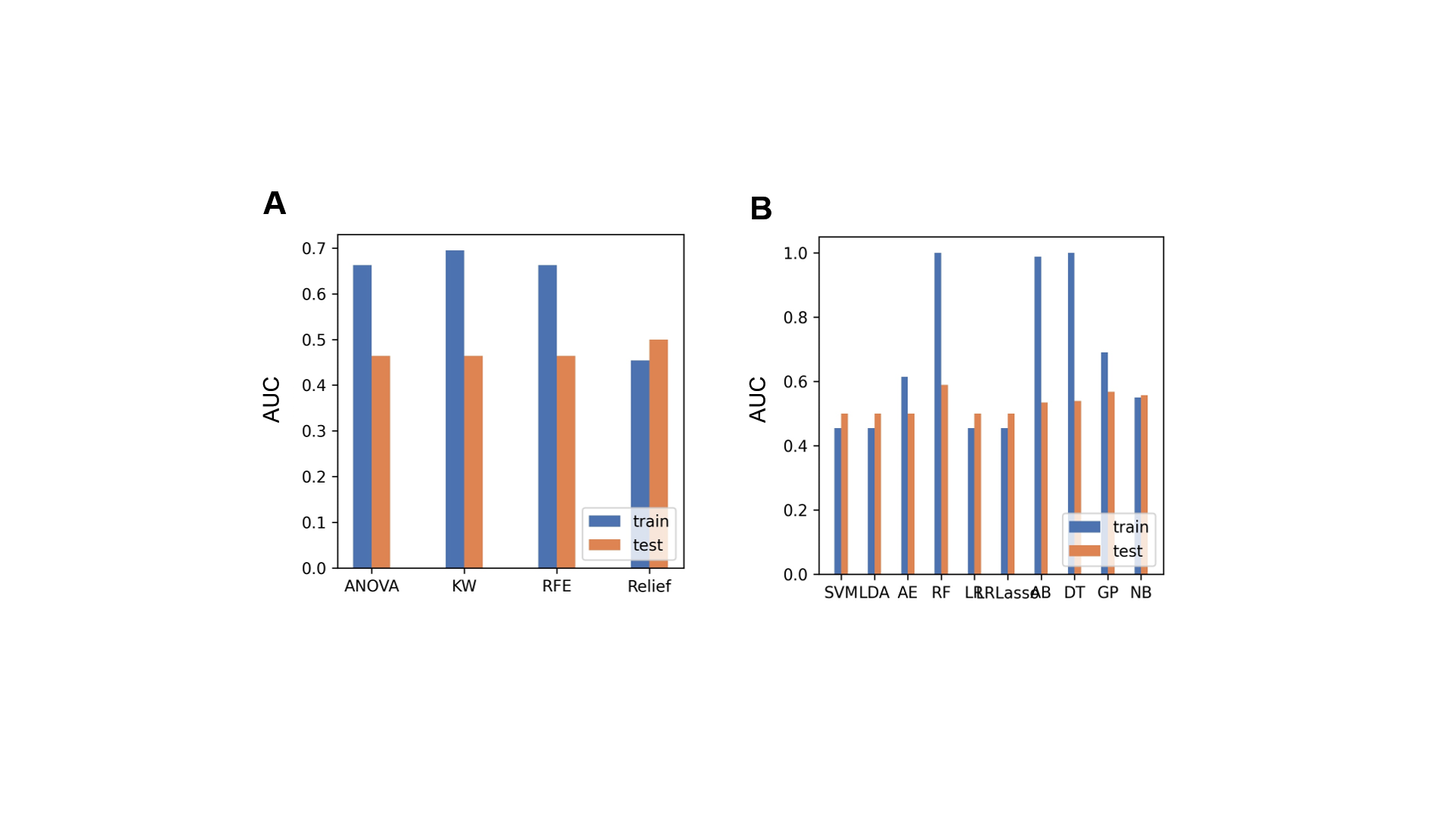

Supplement: Supplementary Figure 4 — The performance of feature selection and the classifier in the immunotherapy response prediction model. The relief algorithm in the feature selection (A) and LR in the classifier (B) had the best performance. [file Image4.tif]

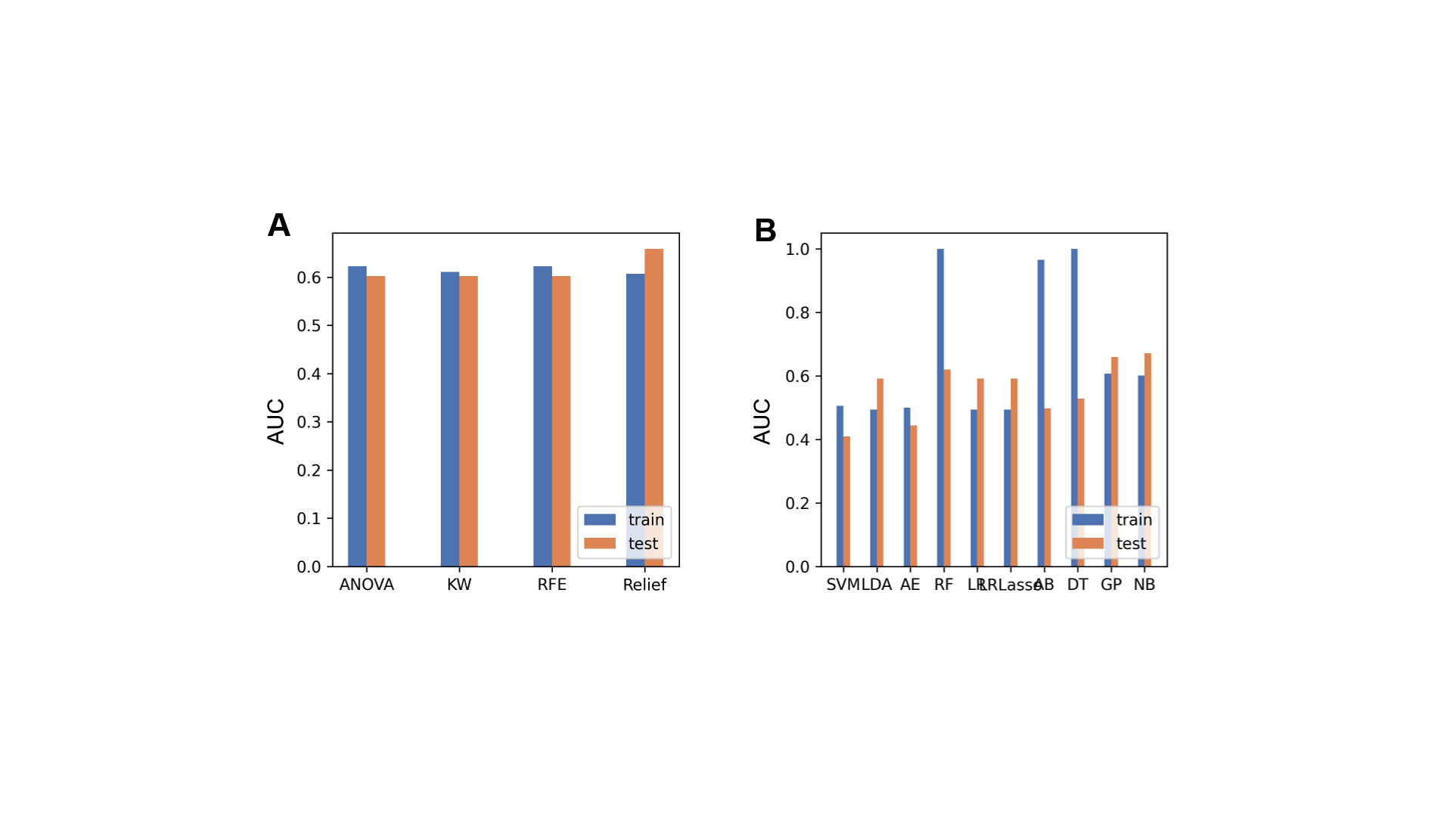

Supplement: Supplementary Figure 5 — The performance of feature selection and the classifier in the PFS prediction model. The relief algorithm in the feature selection (A) and GP in the classifier (B) had the best performance. [file Image5.tif]
