# Supplementary material for: The NSCLC immunotherapy response predicted by tumor-infiltrating T cells via a non-invasive radiomic approach
Source: Front Immunol. 2024 Sep 9;15:1379812. doi: 10.3389/fimmu.2024.1379812 (PMC11416977; doi:10.3389/fimmu.2024.1379812)
Supplement: Supplementary Figure 6 — The FAE panel. The main panel (A), feature preprocessing window (B), model exploration window (C), and result visualization window (D) of FeAture Explorer V.0.5.5. [file Image6.pdf]

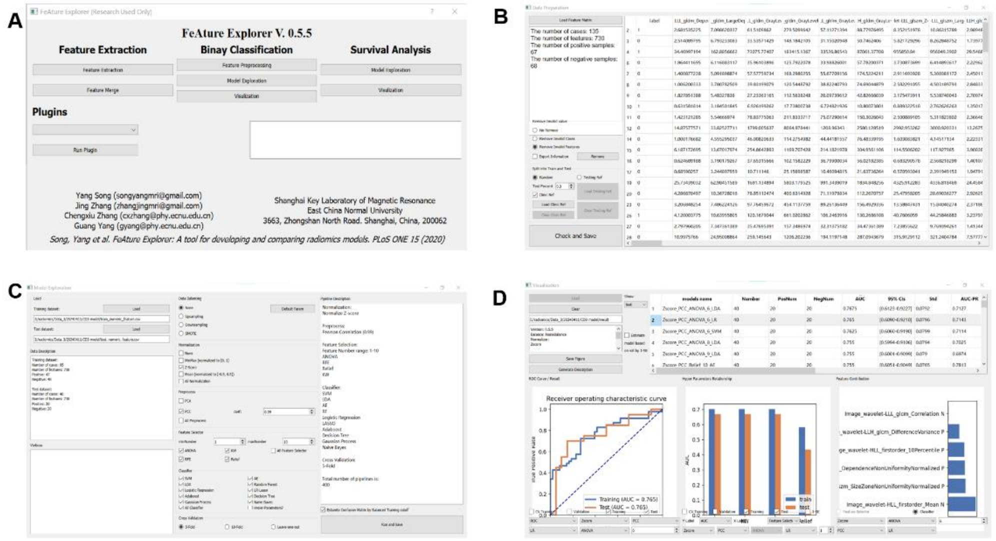

Figure S6. The panel of FAE.

The main panel(A), feature preprocessing window(B), model exploration window(C) and result visualization window (D) of FeAture Explorer V.0.5.5.
